# G-Aligner: a graph-based feature alignment method for untargeted LC–MS-based metabolomics

**DOI:** 10.1186/s12859-023-05525-4

**Published:** 2023-11-14

**Authors:** Ruimin Wang, Miaoshan Lu, Shaowei An, Jinyin Wang, Changbin Yu

**Affiliations:** 1 Fudan University, Shanghai, 200433 Shanghai China; 2 School of Engineering, Westlake University, Hangzhou, 310030 Zhejiang China; 3 Shandong First Medical University and Shandong Academy of Medical Sciences, Jinan, 250021 Shandong China; 4 Zhejiang University, Hangzhou, 310058 Zhejiang China; 5 School of Life Sciences, Westlake University, Hangzhou, 310030 Zhejiang China

**Keywords:** LC–MS, Feature alignment, Multidimensional assignment problem, Combinatorial optimization

## Abstract

**Background:**

Liquid chromatography–mass spectrometry is widely used in untargeted metabolomics for composition profiling. In multi-run analysis scenarios, features of each run are aligned into consensus features by feature alignment algorithms to observe the intensity variations across runs. However, most of the existing feature alignment methods focus more on accurate retention time correction, while underestimating the importance of feature matching. None of the existing methods can comprehensively consider feature correspondences among all runs and achieve optimal matching.

**Results:**

To comprehensively analyze feature correspondences among runs, we propose G-Aligner, a graph-based feature alignment method for untargeted LC–MS data. In the feature matching stage, G-Aligner treats features and potential correspondences as nodes and edges in a multipartite graph, considers the multi-run feature matching problem an unbalanced multidimensional assignment problem, and provides three combinatorial optimization algorithms to find optimal matching solutions. In comparison with the feature alignment methods in OpenMS, MZmine2 and XCMS on three public metabolomics benchmark datasets, G-Aligner achieved the best feature alignment performance on all the three datasets with up to 9.8% and 26.6% increase in accurately aligned features and analytes, and helped all comparison software obtain more accurate results on their self-extracted features by integrating G-Aligner to their analysis workflow. G-Aligner is open-source and freely available at https://github.com/CSi-Studio/G-Aligner under a permissive license. Benchmark datasets, manual annotation results, evaluation methods and results are available at https://doi.org/10.5281/zenodo.8313034

**Conclusions:**

In this study, we proposed G-Aligner to improve feature matching accuracy for untargeted metabolomics LC–MS data. G-Aligner comprehensively considered potential feature correspondences between all runs, converting the feature matching problem as a multidimensional assignment problem (MAP). In evaluations on three public metabolomics benchmark datasets, G-Aligner achieved the highest alignment accuracy on manual annotated and popular software extracted features, proving the effectiveness and robustness of the algorithm.

**Supplementary Information:**

The online version contains supplementary material available at 10.1186/s12859-023-05525-4.

## Background

Liquid chromatography–mass spectrometry (LC–MS) is widely used in the discovery of unknown compounds in untargeted metabolomics [1, 2] In an analysis of multiple LC–MS runs, features of each run are first detected and quantified from raw data by feature extraction algorithms, and features of multiple related LC–MS runs are then combined into one consensus result map by feature alignment algorithms [3]. The accuracy of feature alignment directly determines the rationality and credibility of subsequent statistical and biological analysis. However, features have nonlinear retention time (RT) drifts between runs due to unavoidable slight changes in chromatogram conditions [4], such as column temperature, column aging and contamination, which greatly increases the difficulty of aligning consensus features.

To perform accurate feature alignment, researchers have proposed dozens of methods in the last decades [3, 5]. The feature alignment methods can be divided into two categories according to the alignment steps, retention time alignment and feature matching. Retention time alignment methods, such as DTW [6], PTW [7] and GTW [8], align retention times of multiple runs to a common space by fitting linear or nonlinear warping functions between runs to correct retention shifts and make features of the same analyte have closer elution time. Feature matching methods group features of different runs to consensus feature groups by distances in m/z and RT dimensions. Each consensus feature group represents the predicted corresponding features of the same analyte.

Most existing methods rely heavily on retention time alignment and only use naive nearest neighbor search in feature matching. The representative one is MZmine2 [9]. After aligning retention time with RANSAC [10], MZmine2 selects a reference run and performs pairwise nearest matching to match features of other runs to the reference. However, retention time alignment methods can only describe the drifting trend between runs but cannot accurately correct the retention time of each feature. The retention time of analytes has different drift trends and offsets according to different physical and chemical properties, and elution order may swap when features of different analytes are sufficiently close. To perform more accurate feature matching, LWBmatch [11, 12] considers feature matching between runs as an unbalanced weighted bipartite matching problem and solves the multi-run feature alignment problem by pairwise feature matching. To solve the feature matching problem more comprehensively, OpenMS [13] and XCMS [14] consider the multi-run feature matching problem a clustering problem. OpenMS finds a cluster of the nearest features for each feature among all runs and then chooses the tightest cluster iteratively to get the feature matching results. XCMS first separates features of all runs by m/z bins, then calculates the feature density curve in RT dimension for each bin, and groups the features according to the RT ranges of peaks in the density curve. However, the OpenMS method uses centralized nearest matching, which is prone to fall into local optimum, and the XCMS grouping is only based on dimensionality reduced RT distribution of features, which leads to lower accuracy and limited performance on feature matchings of close analytes.

**Fig. 1:**
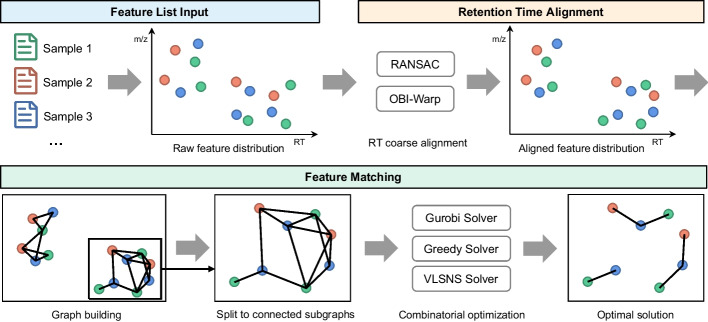
The workflow of G-Aligner. G-Aligner performs feature alignment in three main steps: feature list input, retention time alignment and feature matching. After loading features of multiple samples, G-Aligner first roughly aligns the retention time of features between samples. Then, in the feature matching step, G-Aligner builds multipartite graphs to represent potential matching relationship among features, and utilizes combinatorial optimization to obtain optimal matchings

To improve feature matching performance, in this paper, we propose G-Aligner (Fig. 1), a graph-based feature alignment method for untargeted LC–MS-based metabolomics. G-Aligner is the first method enabling non-centric comprehensive analysis of all potential feature correspondences among multiple LC–MS runs. In the retention time alignment stage, G-Aligner uses the existing RANSAC [10] and OBI-Warp [15] algorithms to perform stable coarse registration between runs. In the feature matching stage, G-Aligner formats the potential correspondences between all features as a multipartite graph, and solves the multi-run feature matching problem as an unbalanced multidimensional assignment problem [16] with three combinatorial optimization methods. In comparison with feature alignment methods in OpenMS, MZmine2 and XCMS, G-Aligner showed significant improvements in feature alignment accuracy and helped all comparison software obtain more accurate results by integrating G-Aligner into their workflow.

## Methods

### Evaluation datasets

We used the two public metabolomics benchmark datasets in [17], named as the TripleTOF 6600 dataset and the QE HF dataset. Both datasets were acquired with the full-scan method. The TripleTOF 6600 dataset was acquired by AB SCIEX TripleTOF 6600 interfaced with Shimazu L30A UPLC, and the QE HF dataset was acquired by Thermo Q Exactive HF with Dionex UltiMate 3000 HPLC. A pair of standard mixtures (SA, SB) were prepared for both datasets, which were composed of seven differential groups of drugs and metabolites (Gd1, Gd2, Gd3, Gm, Gd4, Gd5, Gd6) prepared at different relative concentration ratios of (1/16, 1/4, 1/2, 1/1, 2/1, 4/1, 16/1) in SB: SA. The TripleTOF 6600 dataset had four replicates for each mixture, with a total of eight runs. The QE HF dataset had five replicates for each mixture, with a total of ten runs. Benchmark libraries were provided along with the datasets, containing 970 and 836 analytes in the TripleTOF 6600 and the QE HF datasets. We further screened the benchmark libraries to remove duplicates and obtained 924 and 835 analytes, respectively. In both benchmark datasets, the retention times of mixed compounds have different drift trends due to large differences in physical and chemical properties (Additional file 1: Fig. S1). Even after retention time alignment across runs, the retention times of analyte features still had wide shifts, which increased the difficulty of feature alignment and made the dataset more suitable for feature alignment evaluation.

Furthermore, to evaluate the performance of feature alignment methods on a bigger dataset, we used the dataset published in Metabolights MTBLS562 [18]. The MTBLS562 dataset was acquired with DDA method and acquired by AB SCIEX TripleTOF 6600 with Agilent 1290 UHPLC system. The MTBLS562 dataset contains 40 LC–MS runs of 40 mice (C57BL/6J strain, SPF level) at 5 different ages (4, 12, 24, 32 to 52 weeks, n=8 in each group). Benchmark library was provided along with the dataset, containing 245 analytes. We further screened the benchmark library to remove duplicates and noises and obtained 207 analytes. Some analytes in the library have low abundance in samples with feature intensity heights on the order of hundreds.

### Input preparation

G-Aligner requires users to provide a list of extracted features for each LC–MS run. G-Aligner supports feature extraction results in csv and tsv formats, which can be exported from untargeted metabolomics software. As a general method, G-Aligner only requires users to provide basic feature information, including m/z, retention time and intensity. In case of using profile-based retention time alignment methods that require raw files (such as OBI-Warp), users need to convert raw files to mzML format by MSConvert [19] or aird format by AirdPro [20] for G-Aligner analysis.

### Retention time alignment

To correct retention time drift and reduce the difficulty of subsequent feature matching, G-Aligner provides a feature-based retention time alignment method RANSAC (RANdom SAmple Consensus) and a profile-based method OBI-Warp (Ordered Bijective Interpolated Warping). RANSAC is an iterative method for fitting a model to data and is robust to outliers. In each iteration, the RANSAC method selects a random subset of points from the input data and fits a linear or nonlinear model to these points. The remaining points are then classified into inliers or outliers based on their consistency with the model. Among the iterations, the best-fitting model with the largest number of inliers is selected as the alignment result. OBI-Warp is an extension of dynamic time warping (DTW) for nonlinear retention time warping. For pairs of runs that need to be aligned, OBI-Warp first uses DTW to analyze the spectral similarity and obtain the optimal bijective warping function by dynamic programming. OBI-Warp combines the warping function output from DTW with piecewise cubic Hermitian interpolation and produces smooth warped functions. G-Aligner implemented OBI-Warp in Python for the first time and separated it as an independent installation package for importing by other Python-based software. These two algorithms were also applied in MZmine2 and XCMS, respectively, and their effectiveness and stability have been proven in long-term use. G-Aligner uses linear RANSAC as the default retention time alignment algorithm because it does not require additional raw file import and is faster and more stable.

### Feature matching

#### Graph building

Instead of matching features iteratively to a real or virtual reference run, G-Aligner comprehensively considers potential feature correspondences between all LC–MS runs and performs combinatorial optimization to obtain optimal solutions. After retention time coarse registration, the corresponding features of each analyte in different runs are relatively close in m/z and retention time coordinates. To find potential correspondences among all runs, G-Aligner superimposes the retention time aligned features in all runs on the same coordinate system consisting of m/z and retention time dimensions, searches neighborhoods for each feature according to the m/z and retention time tolerances set by the user, and builds an undirected relation graph containing features of all runs. Each node in the graph represents a feature, and each edge represents a potential feature correspondence between neighbor features.

#### Graph splitting

After graph building, spatially close feature nodes are connected into many connected components, which are maximal connected subgraphs of the relation graph. A connected component is the largest set of potentially corresponding features for each contained analyte and can be treated as the smallest computing unit for combinatorial optimization. G-Aligner splits the relation graph into connected components and performs combinatorial optimization on each connected component to divide and conquer the feature matching problem. The edge weights in each connected component are calculated by the weighted sum of normalized differences in m/z, retention time and intensity. The m/z and retention time differences are normalized by user-specified m/z and retention time tolerances, and the intensity difference is the deviation in intensity normalized by the largest feature intensity in the same connected component in each run. The edge weight is calculated as Eq. 1 and Eq. 2:

1 $$\begin{aligned} normed\ area= & {} \frac{area}{maximum\ area\ in\ the\ partite} \end{aligned}$$

2 $$\begin{aligned} edge\ weight= & {} \frac{1}{3} \times \left( \frac{|\Delta m/z|}{m/z\ tolerance} + \frac{|\Delta RT|}{RT\ tolerance} + \left( 1-\frac{\min (normed\ area)}{\max (normed\ area)} \right) \right) \end{aligned}$$

#### Assignment solving

In graph theory, a multipartite graph is a type of graph in which the nodes can be divided into multiple independent sets. Each set of nodes is called a partite, and each edge connects a pair of nodes from different partites. After graph splitting, each connected component is a multipartite graph where edges do not connect feature nodes of the same run but different runs. To find the optimal correspondence solution, G-Aligner treats the feature matching problem on the multipartite subgraph as an unbalanced multidimensional assignment problem (MAP), in which the feature number differs in partites. MAP is a fundamental combinatorial optimization problem to find optimal matchings in a weighted multipartite graph, in which the sum of weights of the edges is minimum.

**Fig. 2:**
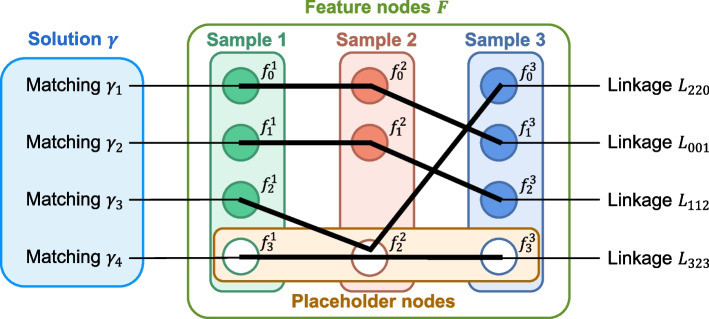
Visual representation of feature nodes, linkages and matchings in a feasible solution

In G-Aligner, we comprehensively considered the neighborhood distribution of features in all runs and defined the unbalanced MAP of the feature matching as follows. For a multipartite graph *G* which contained feature nodes *F* from *M* runs (Eq. 3), let *F*(*k*) represent the feature set of the *k*-th LC–MS run, and $f^k_{ik}$ represent the $i_k$-th feature node in the *k*-th LC–MS run (Eq. 4). *F*(*k*) contains $n_k$ deduplicated feature nodes $\{f^k_0,\dots ,f^k_{n_k-1}\}$ and a placeholder node $f^k_{n_k}$. We define a linkage (M-partite matching) of features as $L_{i_1 \dots i_M}$ (Eq. 5), which contains one feature node from each run. Each linkage represents a possible combination of related features of an analyte. When linkage $L_{i_1 \dots i_M}$ contains a placeholder node $f^k_{n_k}$, it means no feature from the *k*-th run was matched in the linkage. Accordingly, we recorded the empty linkage containing placeholder nodes of all runs as $L_{n_1 \dots n_M}$. The feasible solutions $\varGamma$ of MAP should satisfy the following constraints (Eq. 6, 7, 8, 9). Equation 6 states that each solution $\gamma$ contains *p* matchings, where *p* is the max node number (containing placeholder node) in each feature set. Equation 7 states that each matching uniquely corresponds to a distinct linkage. Equation 8 states that different matchings in the same solution do not contain shared non-placeholder feature nodes. Equation 9 states that each feasible solution contains all feature nodes. A figure of feature nodes, linkages and matchings in a feasible solution is shown in Fig. 2.

3 $$\begin{aligned} F= & {} \{F(1), \ldots , F(M)\} \end{aligned}$$

4 $$\begin{aligned} F(k)= & {} \left\{ f_{i_k}^k \mid i_k=0, \ldots , n_k\right\} , \text{ for } k=1, \ldots , M \end{aligned}$$

5 $$\begin{aligned} L_{i_1 \ldots i_M}= & {} \left\{ f_{i_1}^1, \ldots , f_{i_M}^M\right\} , \text{ for } i_k=0, \ldots , n_k, k=1, \ldots , M \end{aligned}$$

6 $$\begin{aligned} \gamma= & {} \left\{ \gamma _1, \ldots , \gamma _p\right\} , p=\max \left( n_1, \ldots , n_M\right) +1 \end{aligned}$$

7 $$\begin{aligned} \forall \gamma _j: \gamma _j= & {} L_{i_1 \ldots i_M} \exists ! {\text {tuple}}\left( i_1, \ldots , i_M\right) \end{aligned}$$

8 $$\begin{aligned}{} & {} \gamma _i \cap \gamma _j \subset L_{n_1 \ldots n_M}, \text{ for } i \ne j \end{aligned}$$

9 $$\begin{aligned}{} & {} F=U_{j=1}^p \gamma _j \end{aligned}$$

In order to measure the quality of solutions, we calculated the cost of each solution and selected the one with the lowest cost as the optimal solution. Let $C_\gamma$ represents the cost of solution $\gamma$, and $C_{\gamma _i}$ represents the cost of matching $\gamma _i$ (Eq. 10). The cost of a solution is the total cost of all matchings contained. $C_{\gamma _i}$ consists of distribution cost $C_{distribution}$, connectivity cost $C_{connectivity}$, missing node cost $C_{missing}$ and full match bonus $B_{full\_match}$ (Eq. 11).

The distribution cost in a matching describes the dispersion of matched nodes. For matching $\gamma _i$, we first extract the subgraph $G_{\gamma _i}$ from the multipartite graph *G*, which contains all feature nodes and interconnected edges in $\gamma _i$. Then, we calculated the minimum spanning tree (MST) of $G_{\gamma _i}$ to find the minimum total weight of the edge subsets connecting all nodes, and used the total weight as the distribution cost $C_{distribution}$ for matching $\gamma _i$ (Eq. 12). In matchings with placeholder nodes and far unconnected clusters, the number of edges in MST is lower than expected $M-1$, resulting in undesired lower distribution cost and affecting matching priorities. Hence, we introduce the missing node cost and the connectivity cost to penalize the missing edges (Eq. 13, 14). In matchings with placeholder nodes, the edge number reduction equals the number of placeholder nodes. We calculate the missing node cost by multiplying the placeholder node number $N_p$ by a penalty factor $w_p$. In far matchings, feature nodes are far apart and form multiple clusters, which results in missing edges between clusters. The missed number of edges is equal to the number of connected subgraphs in $G_{\gamma _i}$. Similarly, we calculate the connectivity cost by multiplying the number of connected subgraphs $N_c$ by a penalty factor $w_c$. Besides, we believe matchings with more feature nodes have greater matching priority. Appropriate incentives should be given to full matchings when competing for nodes with others. For connected full matchings, we introduce a bonus factor *b* and subtract it from the matching cost (Eq. 15). All factors are optimized as built-in parameters and do not require user modification.

10 $$\begin{aligned} C_\gamma= & {} \sum _{j=1}^p C_{\gamma _j} \end{aligned}$$

11 $$\begin{aligned} C_{\gamma _j}= & {} C_{distribution}+C_{connectivity}+C_{missing}-B_{full\_match} \end{aligned}$$

12 $$\begin{aligned} C_{distribution}= & {} \sum _{e \in E(T)} W(e), T={\text {MST}}\left( G_{\gamma _i}\right) \end{aligned}$$

13 $$\begin{aligned} C_{connectivity}= & {} w_c * N_c \end{aligned}$$

14 $$\begin{aligned} C_{missing}= & {} w_p * N_p \end{aligned}$$

15 $$\begin{aligned} B_{full\_match}= & {} b \end{aligned}$$

However, the multidimensional assignment problem is a well-known NP-Hard problem at three or higher dimensions. There is no known algorithm for finding the optimal solution in polynomial time. To solve MAP efficiently, G-Aligner proposed three solvers: the Gurobi solver, the Greedy solver and the VLSNS solver.

The Gurobi solver converts the multidimensional assignment problem to an integer linear programming problem and uses the state-of-the-art commercial Gurobi optimizer to find the global optimal solution. By inputting matching costs, feasible solution rules and optimization objectives into the model, the Gurobi optimizer can efficiently calculate optimization results, much faster than other linear programming solvers such as the OR-Tools developed by Google. The pseudocode of the Gurobi solver is detailed in Additional file 1: Algorithm S1.

The Greedy solver pays more attention to the quality of a single matching than the solution. In each iteration, the Greedy solver finds the minimum cost matching, adds the matching to the solution, and sets the corresponding costs of matched features to infinite to avoid generating infeasible solutions. In this way, the Greedy solver does not sacrifice the best matchings for a lower cost of solutions. The pseudocode of the Greedy solver is detailed in Additional file 1: Algorithm S2.

However, the Gurobi and Greedy solver must compute the matching costs for all permutations before running. For faster calculations, we propose the VLSNS solver to solve the unbalanced MAPs, which calculates costs as needed during optimization. The VLSNS solver used a recent metaheuristic approach, known as the very large-scale neighborhood search (VLSNS) [21], which finds near-optimal solutions to the MAP by iteratively transforming the current solution into a better solution in the neighborhood. The VLSNS solver converts the multidimensional assignment problem to linear (two-dimensional) assignment problems (LAP) between each partite and the others. In each iteration, the VLSNS solver finds an optimal permutation for each partite and updates the solution permutation in the best-improved partite until no lower cost can be obtained. The pseudocode of the VLSNS solver is detailed in Additional file 1: Algorithm S3.

Since the solution space is not fully searched, VLSNS is not guaranteed to obtain global optimal results and is prone to fall into local optimum. As compensation, multiple start solutions are provided to the solver for parallel optimization, and the optimized solution with the lowest cost is used as the final result. We provide two solution initialization methods: the MSR method (Additional file 1: Algorithm S4.1) and the MSG method (Additional file 1: Algorithm S4.2). The MSR (multi-solution random) method generates multiple solutions at random. The MSG (multi-solution grid) method generates random solutions first and then equidistantly rolls the permutation order in each partite into multiple grid solutions for each random solution. By adopting the multi-start strategy, the VLSNS solver ensures the computation accuracy and is able to obtain near global optimal results while having a faster calculation speed.

### Big graph acceleration

In cases of dense feature distribution and insufficient accuracy of retention time alignment, some connected components may contain a large number of nodes. As the number of nodes increases, the computing time increases polynomially. For efficient analysis of big graphs, we proposed a graph segmentation method to divide big graphs into multiple small subgraphs, and a result merging method to combine solutions of small graphs to a final result. The graph splitting method iteratively divides the graph into two subgraphs by LC–MS run according to the preset node number limit. Nodes in runs with adjacent acquisition time are segmented into the same subgraphs, and the total number of nodes in the two subgraphs is similar. After splitting, G-Aligner performs the optimization methods described above to obtain optimal solutions for each subgraph. Then, G-Aligner assembles the solutions of multiple subgraphs with the result merging method. The result merging method first computes the minimum weight of the edges between each pair of matchings as their distance. Then, matchings are iteratively merged in ascending order of distance when they do not contain nodes from the same LC–MS run.

Besides, G-Aligner can be recursively executed to meet diverse alignment requirements. By default, G-Aligner scans the directory of the feature extraction result folder provided by the user and recursively aligns the results in each subfolder from bottom to top according to the folder structure. For example, in the scenario where each sample has multiple technical replicates, users can put all replicates of each sample in the same subfolder, so that G-Aligner will first align the technical replicates of each sample, and then align samples according to the mean feature m/z and retention time among replicates. Optionally, this recursive align method provides more sample relation information for the algorithm, facilitating better results and faster analysis in large cohort alignments.

## Results

### Evaluation workflow

We compared G-Aligner with the feature alignment methods in three popular untargeted metabolomics software: MZmine2 (version 2.53), OpenMS (version 2.7.0), and XCMS (version 3.18.0), as representatives of local nearest matching of paired runs, multi-run centric matching and multi-run non-centric matching respectively, on the TripleTOF 6600 dataset, the QE HF dataset and the MTBLS562 dataset. Moreover, since we had problems running LWBmatch, we implemented a local bipartite solver for comparison, as a representative of bipartite pairwise matching methods. After selecting the LC–MS run with the most features as a reference run, the local bipartite solver matched other LC–MS runs iteratively in descending order of feature numbers to the reference run. The local bipartite solver treats feature matching as a weighted bipartite assignment problem and uses a modified Jonker-Volgenant algorithm [22] to find the maximum cardinality matching with a minimum sum of matching distances.

In evaluations of G-Aligner and other methods, we first manually annotated features of library analytes for each dataset and conducted a comprehensive assessment of the feature alignment algorithms on the same manually annotated feature set to exclude differences in feature extraction. Then, we integrated G-Aligner into the workflow of each software to evaluate the improvement on their self-extracted features. In parameter settings, we first manually selected a set of reasonable parameters based on the distribution of the data, and then manually fine-tuned them to obtain the best parameters to achieve the highest detection rate and alignment performance of features corresponding to the compounds in the library. The fine-tuned parameters of compared software were summarized in Additional file 1: Tables S1, S2 and S3. Notably, the differences in feature extraction parameter settings may lead to different distributions on extracted features and result in different difficulties in feature alignment. However, the parameter settings for feature extraction are less important to the evaluation results, we only need to evaluate G-Aligner and other methods on the same set of feature extraction results under the same alignment difficulty.

### Evaluation on manually annotated features

To perform a comprehensive evaluation for feature alignment algorithms, all algorithms should be evaluated on the same set of features to control the variable in feature distribution. To create a feature dataset with standard alignment results for each dataset, we extracted and annotated features of library analytes with the MetaPro [23] batch inspection tool. For each analyte in library, we extracted features close to the m/z and RT coordinates in each run, in which at most one feature was manually annotated as the corresponding feature. All of the extracted nearby features were added to the feature datasets. Each feature was represented by its apex m/z (the apex m/z of the spectral peak at apex RT), apex RT and integrated area. The annotation procedure was detailed in Additional file 1: Appendix S1.

We utilized the annotated results as references to evaluate the accuracy of feature alignment algorithms. When importing the manually annotated features into the comparison software, we directly accessed the source code of the compared software in Python, Java, and R environments, and assembled or re-extracted the features into supported memory objects, since all comparison software lacked external feature import capabilities or only supported specific feature formats. The m/z and retention time ranges of each feature used in re-extraction were kept the same as MetaPro extracted features, which ensured the consistency of evaluation datasets.

**Fig. 3:**
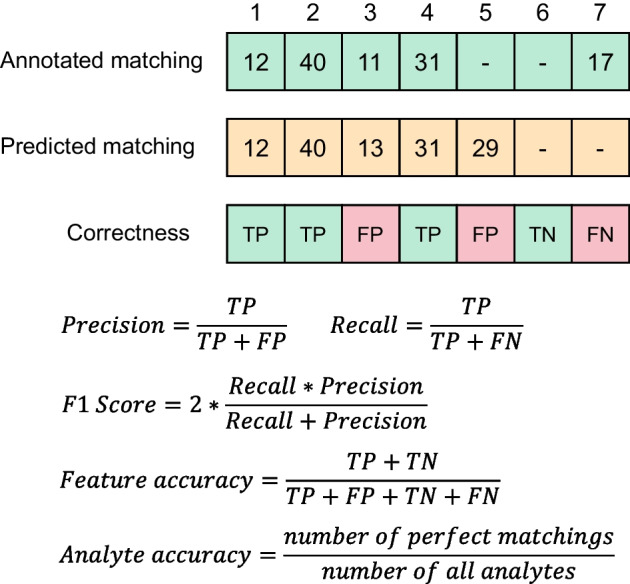
Definitions of evaluation metrics. In the example matching of 7 samples, numbers in matching vectors are the index numbers of matched features in the feature lists of corresponding samples

To benchmark the performance of the feature alignment, we compare the predicted matchings with the manual annotation results (Fig. 3). For each analyte, the predicted matching with the most common features with the annotated matching was considered its corresponding matching, while the contained features were considered its corresponding features. The corresponding feature of each analyte in each sample may differ between the predicted and annotated matchings. For each analyte, the alignment in a sample was considered true positive (TP) when the corresponding feature was present in both aligned and annotated results, false positive (FP) when the corresponding feature was only present in the aligned results, true negative (TN) when there was no corresponding feature in the aligned and annotated results, and false negative (FN) when the corresponding feature was only present in the annotated results. Then, we counted the feature alignment status of all compared methods and calculated the precision (P), recall (R), F1 score (F), feature accuracy (F_ACC), and analyte accuracy (A_ACC) accordingly on each dataset.

In evaluation of the feature alignment accuracy, we compared G-Aligner with other methods on the manually annotated features of each dataset and summarized the results in Table 1, 2, 3. The combinatorial optimization methods in G-Aligner outperformed other comparison methods and achieved the best performance on all datasets with the most TP and FP and the least FP and FN, proving the necessity of multi-run analysis. Among the combinatorial optimization solvers, the Greedy solver obtained less accurate results than the Gurobi solver and VLSNS solver. The Greedy solver focused only on local optimal matchings and ignored corresponding suboptimal matches in the greedy search, which illustrates the importance of global optimization. The Gurobi solver and VLSNS solver were too slow and were skipped in evaluations of the MTBLS562 dataset. G-Aligner achieved the best result on all datasets, with F1 scores at 0.999, 0.998 and 1.000, feature accuracy at 0.999, 0.997 and 0.999, and analyte accuracy at 0.990, 0.989 and 0.981, respectively, in the TripleTOF 6600, QE HF and MTBLS562 dataset. Compared to the best-performed OpenMS method, G-Aligner achieved 1.1%, 1.9% and 0.6% higher feature accuracy and 5.3%, 8.7% and 14.0% higher analyte accuracy, respectively.

**Table 1:** Evaluation results on manually annotated feature sets of the TripleTOF 6600dataset

	TP	FP	TN	FN	P	R	F	F_ACC	A_ACC
MZmine2 RANSAC	7163	77	0	152	0.989	0.979	0.984	0.969	0.883
OpenMS QT	7303	64	0	25	0.991	0.997	0.994	0.988	0.937
XCMS Group	6916	228	0	248	0.968	0.965	0.967	0.936	0.837
XCMS OBI-Warp	6797	253	0	342	0.964	0.952	0.958	0.920	0.777
Local bipartite	7150	75	0	167	0.990	0.977	0.983	0.967	0.918
G-Aligner Greedy	7381	4	0	7	0.999	0.999	**0.999**	**0.999**	**0.990**
G-Aligner Gurobi	7382	3	0	7	1.000	0.999	**0.999**	**0.999**	**0.990**
G-Aligner VLSNS_MSR	7382	3	0	7	1.000	0.999	**0.999**	**0.999**	**0.990**
G-Aligner VLSNS_MSG	7382	3	0	7	1.000	0.999	**0.999**	**0.999**	**0.990**

The results with the highest performance in the comparison are indicated in bold

**Table 2:** Evaluation results on manually annotated feature sets of the QE HF dataset

	TP	FP	TN	FN	P	R	F	F_ACC	A_ACC
MZmine2 RANSAC	7982	103	0	265	0.987	0.968	0.977	0.956	0.831
OpenMS	8167	146	0	37	0.982	0.995	0.989	0.978	0.902
XCMS Group	6812	629	0	909	0.915	0.882	0.899	0.816	0.715
XCMS OBI-Warp	6749	677	0	924	0.909	0.880	0.894	0.808	0.667
Local bipartite	8019	78	0	253	0.990	0.969	0.980	0.960	0.884
G-Aligner Greedy	8320	12	0	18	0.999	0.998	**0.998**	0.996	**0.989**
G-Aligner Gurobi	8323	7	0	20	0.999	0.998	**0.998**	**0.997**	**0.989**
G-Aligner VLSNS_MSR	8323	7	0	20	0.999	0.998	**0.998**	**0.997**	**0.989**
G-Aligner VLSNS_MSG	8323	7	0	20	0.999	0.998	**0.998**	**0.997**	**0.989**

The results with the highest performance in the comparison are indicated in bold

**Table 3:** Evaluation results on manually annotated feature sets of the MTBLS562 dataset

	TP	FP	TN	FN	P	R	F	F_ACC	A_ACC
MZmine2 RANSAC	7822	25	0	433	0.997	0.948	0.972	0.945	0.744
OpenMS	8221	34	0	25	0.996	0.997	0.996	0.993	0.841
XCMS Group	6115	847	0	1318	0.878	0.823	0.850	0.739	0.145
XCMS OBI-Warp	6230	868	0	1182	0.878	0.841	0.859	0.752	0.169
Local bipartite	8173	1	0	106	1.000	0.987	0.993	0.987	0.937
G-Aligner Greedy	8269	3	0	8	1.000	0.999	0.999	**0.999**	0.976
G-Aligner Gurobi	8272	3	0	5	1.000	0.999	**1.000**	**0.999**	**0.981**
G-Aligner VLSNS_MSR	8272	3	0	5	1.000	0.999	**1.000**	**0.999**	**0.981**
G-Aligner VLSNS_MSG	8272	3	0	5	1.000	0.999	**1.000**	**0.999**	**0.981**

The results with the highest performance in the comparison are indicated in bold

### Evaluation on software self-extracted features

Since the distribution of all untargeted extracted features was more complicated than the manually annotated feature datasets, we further evaluated the performance of G-Aligner on full untargeted extracted features. Considering that there may be implicit correlations between the alignment methods and their analysis pipeline, we integrated G-Aligner into the workflow of each software and compared G-Aligner with their original alignment methods in the native environment. To obtain benchmarks for alignment, we used the manually annotated results to infer the correct alignment of untargeted extracted features for each software. Since the feature of each analyte corresponds to the same LC–MS signal in each file, the corresponding features extracted by different software should be extremely near. We matched the untargeted extracted features to the manually annotated features with m/z and retention time tolerances at (0.01Da, 0.1min) on the TripleTOF 6600 dataset, (0.005Da, 0.1min) on the QE HF dataset and (0.015Da, 0.1min) on the MTBLS562 dataset. If more than one feature was matched within the tolerances, the closest was selected as the inferred corresponding feature. The distance between features was the squared sum of m/z and retention time deviations normalized by the inverse of the tolerance. To benchmark the feature alignment performance on untargeted extracted features, we used the same evaluation metrics as on manually annotated features by matching alignment results to inferred annotation results. For each software, we compared G-Aligner with the native feature alignment methods on the alignment performance of all analytes on all datasets, including match status, precision, recall, F1 score, feature accuracy and analyte accuracy.

In evaluation results on untargeted extracted features of the three software (Tables 4, 5, 6), all evaluation methods achieved less accurate results than on the manually annotated features, which was mainly due to the increase in complexity in feature distributions. Different from manually annotated data, false detections and missing detections always happens in untargeted feature extraction, causing more interference to the alignment algorithms. In the evaluation of software self-extracted features, the combinatorial optimization methods in G-Aligner achieved obviously better performance than local pairwise and native methods of all software on all datasets. G-Aligner proved its robustness on untargted extracted features of the three software with different qualities; OpenMS had the most missing features, MZmine2 had fewer, and XCMS had the least. The Gurobi and VLSNS solver achieved the best performance in most feature sets, but was less accurate than the Greedy solver on the OpenMS features of the TripleTOF 6600 dataset. Although the Greedy solver only performs local optimization and always inferior to the Gurobi and VLSNS solver in most cases, the strategy of finding local optimum matching could be less disturbed for feature sets with insufficient quality. In most evaluations on the complex distributed untargted extracted features, the VLSNS solver with the MSG solution initialization method showed higher accuracy than with the MSR method, which indicated that the MSR method might generate less dispersed initial solutions compared due to excessive dependence on randomness, and it was more prone to lead the VLSNS solver into suboptimal solutions. G-Aligner achieved the best performance on untargeted extracted features on all datasets, with 1.7%, 9.0% and 0.5% higher feature accuracy, 5.8%, 26.6% and 7.7% higher analyte accuracy than MZmine2, 2.4%, 1.5% and 1.7% higher feature accuracy, 13.5%, 10.6% and 18.4% higher analyte accuracy than OpenMS, 9.8%, 2.6% and 9.4% higher feature accuracy, 24.5%, 11.4% and 24.7% higher analyte accuracy than XCMS, respectively on the TripleTOF 6600, QE HF and MTBLS562 dataset.

**Table 4:** Evaluation results on software self-extracted features on the TripleTOF 6600 dataset

	TP	FP	TN	FN	P	R	F	F_ACC	A_ACC
MZmine2 RANSAC	6716	129	422	125	0.981	0.982	0.981	0.966	0.869
Local bipartite	6688	122	413	169	0.982	0.975	0.979	0.961	0.882
G-Aligner Greedy	6847	98	403	44	0.986	0.994	0.990	0.981	0.925
G-Aligner Gurobi	6859	84	403	46	0.988	0.993	**0.991**	0.982	0.926
G-Aligner VLSNS_MSR	6846	89	403	54	0.987	0.992	0.990	0.981	0.922
G-Aligner VLSNS_MSG	6862	81	403	46	0.988	0.993	**0.991**	**0.983**	**0.927**
OpenMS QT	5771	336	1204	81	0.945	0.986	0.965	0.944	0.741
Local bipartite	5355	338	1248	451	0.941	0.922	0.931	0.893	0.697
G-Aligner Greedy	5922	140	1230	100	0.977	0.983	**0.980**	**0.968**	0.874
G-Aligner Gurobi	5910	152	1233	97	0.975	0.984	0.979	0.966	**0.876**
G-Aligner VLSNS_MSR	5906	156	1233	97	0.974	0.984	0.979	0.966	0.873
G-Aligner VLSNS_MSG	5910	152	1233	97	0.975	0.984	0.979	0.966	**0.876**
XCMS Group	6567	251	38	536	0.963	0.925	0.943	0.894	0.712
XCMS OBI-Warp	6173	293	39	887	0.955	0.874	0.913	0.840	0.600
Local bipartite	6939	152	51	250	0.979	0.965	0.972	0.946	0.866
G-Aligner Greedy	7281	52	47	12	0.993	0.998	**0.996**	0.991	0.956
G-Aligner Gurobi	7283	47	47	15	0.994	0.998	**0.996**	**0.992**	**0.957**
G-Aligner VLSNS_MSR	7270	51	47	24	0.993	0.997	0.995	0.990	0.951
G-Aligner VLSNS_MSG	7283	47	47	15	0.994	0.998	**0.996**	**0.992**	**0.957**

The results with the highest performance in the comparison are indicated in bold

**Table 5:** Evaluation results on software self-extracted features on the QE HF dataset

	TP	FP	TN	FN	P	R	F	F_ACC	A_ACC
MZmine2 RANSAC	6995	11	566	778	0.998	0.900	0.947	0.906	0.721
Local bipartite	7705	6	563	76	0.999	0.990	0.995	0.990	0.975
G-Aligner Greedy	7751	5	563	31	0.999	0.996	**0.998**	**0.996**	**0.987**
G-Aligner Gurobi	7751	5	563	31	0.999	0.996	**0.998**	**0.996**	**0.987**
G-Aligner VLSNS_MSR	7746	5	563	36	0.999	0.995	0.997	0.995	0.986
G-Aligner VLSNS_MSG	7751	5	563	31	0.999	0.996	**0.998**	**0.996**	**0.987**
OpenMS QT	7039	20	1166	125	0.997	0.983	0.990	0.983	0.887
Local bipartite	7092	8	1169	81	0.999	0.989	0.994	0.989	0.972
G-Aligner Greedy	7161	2	1169	18	1.000	0.997	**0.999**	**0.998**	**0.993**
G-Aligner Gurobi	7161	2	1169	18	1.000	0.997	**0.999**	**0.998**	**0.993**
G-Aligner VLSNS_MSR	7161	2	1169	18	1.000	0.997	**0.999**	**0.998**	**0.993**
G-Aligner VLSNS_MSG	7161	2	1169	18	1.000	0.997	**0.999**	**0.998**	**0.993**
XCMS Group	7934	99	134	183	0.988	0.977	0.983	0.966	0.846
XCMS OBI-Warp	7940	70	135	205	0.991	0.975	0.983	0.967	0.846
Local bipartite	8057	36	148	109	0.996	0.987	0.991	0.983	0.938
G-Aligner Greedy	8141	15	148	46	0.998	0.994	**0.996**	**0.993**	**0.960**
G-Aligner Gurobi	8141	15	148	46	0.998	0.994	**0.996**	**0.993**	**0.960**
G-Aligner VLSNS_MSR	8131	20	148	51	0.998	0.994	**0.996**	0.991	0.957
G-Aligner VLSNS_MSG	8141	15	148	46	0.998	0.994	**0.996**	**0.993**	**0.960**

The results with the highest performance in the comparison are indicated in bold

**Table 6:** Evaluation results on software self-extracted features on the MTBLS562 dataset

	TP	FP	TN	FN	P	R	F	F_ACC	A_ACC
MZmine2 RANSAC	5751	106	2398	25	0.982	0.996	0.989	0.984	0.768
Local bipartite	5719	58	2448	55	0.990	0.990	0.990	0.986	0.845
G-Aligner VLSNS_MSR	5744	67	2435	34	0.988	0.994	0.991	0.988	**0.850**
G-Aligner VLSNS_MSG	5758	66	2434	22	0.989	0.996	**0.992**	**0.989**	0.845
OpenMS QT	3916	139	4182	43	0.966	0.989	0.977	0.978	0.715
Local bipartite	3933	32	4277	38	0.992	0.990	0.991	0.992	0.894
G-Aligner VLSNS_MSR	3961	34	4275	10	0.991	0.997	0.994	**0.995**	**0.899**
G-Aligner VLSNS_MSG	3966	35	4275	4	0.991	0.999	**0.995**	**0.995**	**0.899**
XCMS Group	5786	386	1671	437	0.937	0.930	0.934	0.901	0.676
XCMS OBI-Warp	5756	437	1671	416	0.929	0.933	0.931	0.897	0.652
Local bipartite	6425	37	1807	11	0.994	0.998	0.996	0.994	**0.923**
G-Aligner VLSNS_MSR	6425	38	1807	10	0.994	0.998	0.996	0.994	0.918
G-Aligner VLSNS_MSG	6431	37	1807	5	0.994	0.999	**0.997**	**0.995**	**0.923**

The results with the highest performance in the comparison are indicated in bold

### Analysis time

We measured the time costs of G-Aligner and all comparison software on a Windows 11 computer with an Intel(R)_Core(TM)_i9-12900KS CPU (Additional file 1: Tables S4, S5). In evaluation of the manually annotated features, G-Aligner took 1.5 min, 3 min, 0.8 min, respectively on the TripleTOF 6600, QE HF, MTBLS562 dataset. In evaluation on software-extracted features, G-Aligner took 0.6 to 3 min per file on the TripleTOF 6600 dataset, 0.4 to 1 min per file on the QE HF dataset and 1 to 8 min per file on the MTBLS562 dataset. Due to the comprehensive analysis on all potential feature correspondences, combinatorial methods in G-Aligner spent more time than other compared methods. Among the solvers in G-Aligner, the VLSNS solver was up to 9.6 times faster than the Gurobi and Greedy solver and empowered G-Aligner to achieve more accurate feature alignment in acceptable time.

### Discussion

In comparison with popular feature alignment methods in OpenMS, MZmine2 and XCMS, all combinatorial optimization methods in G-Aligner showed significant improvement on the manually annotated and untargeted extracted features of all datasets. The solvers of G-Aligner had different optimization strategies. The Greedy solver is a local optimization method that finds minimum cost matchings iteratively, and the Gurobi and VLSNS solvers aim to find a global optimal solution that minimizes the sum of matching costs. The Gurobi solver and the VLSNS solver achieved equal or better feature alignment accuracy than the Greedy solver in most comparisons, proving the general superiority of global optimization. In global optimization methods, the Gurobi solver treated the MAPs as integer linear programming problems and guaranteed to find global optimal results, while the VLSNS solver was designed to find near-optimal results in less time. With the assistance of the MSG solution initialization method, the VLSNS solver achieved the same global optimal results as the Gurobi solver in all evaluations with less computation time. As a general feature alignment method, G-Aligner helped all evaluated software achieve more accurate feature alignment results and proved its generality and robustness.

## Conclusion

In this study, we proposed G-Aligner to improve feature matching accuracy for untargeted metabolomics LC–MS data. G-Aligner considered features of all runs as graph nodes and potential correspondences between features as edges. By treating the feature matching problem as multidimensional assignment problems on multipartite graphs, G-Aligner achieved non-centric analysis of all potential correspondences between features of all runs for the first time. Due to the comprehensive analysis of feature distribution, G-Aligner showed obvious advantages in feature matching accuracy. Compared to popular methods, G-Aligner achieved the highest feature alignment accuracy on all benchmark datasets with reasonable computational time.

The main limitation of G-Aligner is the lack of computing speed. To comprehensively consider all potential feature correspondences, G-Aligner modeled the feature matching problem to a multidimensional assignment problem, which was NP-Hard and inevitably requires a lot of computing time. Although we provided two acceleration methods to keep the running time within an acceptable range, there was still a gap between painless application. There are two ways to promote the comprehensive analysis of all potential feature correspondences. The first way is to introduce GPU acceleration to speed up the parallel calculations in G-Aligner. Another way is to go down to the basis principle of retention time drifting. Analyzing massive amount of potential correlations is inevitable in phenomena analysis. Instead, using the phenomena data to estimate the principle of drifting such as retention modeling may be a new efficient way for untargted feature alignment. Furthermore, G-Aligner will be integrated into MetaPro for feature alignment in the untargeted analysis module.

### Supplementary Information

**Additional file 1.** Supplementary information of this study; **Algorithm S1**, Pseudocode of the Gurobi solver; **Algorithm S2**, Pseudocode of the Greedy solver; **Algorithm S3**, Pseudocode of the VLSNS solver; **Algorithm S4**, Pseudocode of the solution initialization methods of the VLSNS solver; **Figure S1**, The RT drift distribution of library analytes on the TripleTOF 6600 dataset and the QE HF dataset; **Table S1**, Optimized parameters used in the TripleTOF 6600 dataset evaluation; **Table S2**, Optimized parameters used in the QE HF dataset evaluation; **Table S3**, Optimized parameters used in the MTBLS562 dataset evaluation; **Table S4**, Time cost on manually annotated feature sets of the TripleTOF 6600 dataset, the QE HF dataset and the MTBLS562 dataset; **Table S5**, Time cost on software self-extracted feature sets of the TripleTOF 6600 dataset, the QE HF dataset and the MTBLS562 dataset; **Appendix S1**. Data annotation procedure in MetaPro.

## Data Availability

Benchmark datasets, manual annotation results, evaluation methods and results are available at https://doi.org/10.5281/zenodo.8313034

## Abbreviations

LC–MS: Liquid chromatography–mass spectrometry
m/z: Mass to charge
RT: Retention rime
RANSAC: RANdom SAmple Consensus
OBI-Warp: Ordered bijective interpolated warping
DTW: Dynamic time warping
MAP: Multidimensional assignment problem
MST: Minimum spanning tree
VLSNS: Very large-scale neighborhood search
LAP: Linear assignment problem
MSR: Multi-solution randoms
MSG: Multi-solution grid
TP: True positive
FP: False positive
TN: True negative
FN: False negative
P: Precision score
R: Recall score
F: F1 score
F_ACC: Feature accuracy
A_ACC: Analyte accuracy

## Code availability

G-Aligner is open-source and freely available at https://github.com/CSi-Studio/G-Aligner under a permissive license.
