# Supplementary material for: G-Aligner: a graph-based feature alignment method for untargeted LC–MS-based metabolomics
Source: BMC Bioinformatics. 2023 Nov 14;24:431. doi: 10.1186/s12859-023-05525-4 (PMC10644574; doi:10.1186/s12859-023-05525-4)
Supplement: Supplementary file 1 — Additional file 1. Supplementary information of this study; Algorithm S1, Pseudocode of the Gurobi solver; Algorithm S2, Pseudocode of the Greedy solver; Algorithm S3, Pseudocode of the VLSNS solver; Algorithm S4, Pseudocode of the solution initialization methods of the VLSNS solver; Figure S1, The RT drift distribution of library analytes on the TripleTOF 6600 dataset and the QE HF dataset; Table S1, Optimized parameters used in the TripleTOF 6600 dataset evaluation; Table S2, Optimized parameters used in the QE HF dataset evaluation; Table S3, Optimized parameters used in the MTBLS562 dataset evaluation; Table S4, Time cost on manually annotated feature sets of the TripleTOF 6600 dataset, the QE HF dataset and the MTBLS562 dataset; Table S5, Time cost on software self-extracted feature sets of the TripleTOF 6600 dataset, the QE HF dataset and the MTBLS562 dataset; Appendix S1. Data annotation procedure in MetaPro. [file 12859_2023_5525_MOESM1_ESM.pdf]

Supplementary Information

G-Aligner: A graph-based feature alignment method for untargeted LC-MS-based metabolomics.

Ruimin Wang, Miaoshan Lu, Shaowei An, Jinyin Wang and Changbin Yu

Algorithm S1. Pseudocode of the Gurobi solver**.**

C is the cost matrix associated with the problem. $C_{i_1 \dots i_M}$ is the cost of corresponding linkage $L_{i_1 \dots i_M}$. X is the allocation matrix associated with the solution. $x_{i_1 \dots i_M}$ equals to 1 when corresponding linkage $L_{i_1 \dots i_M}$ is selected as a matching.

Algorithm S1 The Gurobi solver

Input $C = \{C_{i_1 \dots i_M} | i_k = 0, \dots, n_k, k = 1, \dots, M\}$
Initialize the Gurobi optimizer
Initialize $X = \{x_{i_1 \dots i_M} | i_k = 0, \dots, n_k, k = 1, \dots, M\}$
for all $m \in \{1, \dots, M\}$ **do**
    for all $j \in \{0, \dots, n_k - 1\}$ **do**
        Add constraint $sum\{x_{i_1 \dots i_M} | i_m = j\} = 1$
    end for
end for
Add constraint $x_{n_1 \dots n_M} = 0$
Set objective as minimum $sum\{x_{i_1 \dots i_M} C_{i_1 \dots i_M}\}$
Run the Gurobi optimizer to get optimized X
Initialize solution $\gamma = \{\ \}$
for all $x_{i_1 \dots i_M} \in X$ **do**
    if $x_{i_1 \dots i_M} = 1$ **then**
        Add matching $(i_1, \dots, i_M)$ to γ
    end if
end for
Output solution γ

Algorithm S2. Pseudocode of the Greedy solver.

C is the cost matrix associated with the problem. $C_{i_1 \dots i_M}$ is the cost of corresponding linkage $L_{i_1 \dots i_M}$. In each iteration, the Greedy solver finds the minimum cost matching $C_{j_1 \dots j_M}$, adds the matching to solution, and sets costs including matched features to infinite to avoid generating infeasible solutions.

Algorithm S2 The Greedy solver

Input $C = \{C_{i_1 \dots i_M} | i_k = 0, \dots, n_k, k = 1, \dots, M\}$
Set $C_{n_1 \dots n_M}$ to infinite
Initialize solution $\gamma = \{\ \}$
Find the minimum cost $C_{j_1 \dots j_M} \leftarrow min\{C\}$
while $C_{j_1 \dots j_M} \neq infinite$ **do**
    Add matching $(j_1, \dots, j_M)$ to γ
    for all $m \in \{1, \dots, M\}$ **do**
        Set $\{C_{i_1 \dots i_M} | i_m = j_m\}$ to infinite
    end for
    Find the minimum cost $C_{j_1 \dots j_M} \leftarrow min\{C\}$
end while
Output solution γ

Algorithm S3. Pseudocode of the VLSNS solver.

n_k is the feature number of the k-th sample in a multipartite subgraph. I is the number of multi-start solutions. After generating I start solutions with input solution initialization method, the VLSNS solver transforms the MAP to multiple LAPs in the neighborhood iteratively, and solve LAPs to find better permutations for each sample column.

Algorithm S3 The VLSNS solver

1: Input $\{n_1, ..., n_M\}$, I, solution initialization method
2: Apply the solution initialization method to generate I initial solutions $\Gamma = \{\gamma^1, ..., \gamma^I\}$
3: **for all** solution $\gamma \in \Gamma$ **do**
4:     **for all** $m \in \{1, ..., M\}$ **do**
5:         Convert the MAP of all samples to the LAP between sample m and other samples
6:         Calculate the solution cost matrix $C_{p \times p}$ for all permutations of column m
7:         Solve the LAP and find the optimal permutation of column m with cost C_m
8:     **end for**
9:     Find the minimum cost C_j in $\{C_1, ..., C_M\}$
10:    **if** $C_j < C_i$ **then**
11:        Apply the optimal permutation of column j to solution γ
12:        Update the solution cost C_γ to C_j
12:        Go to step 4
15:    **end if**
16:    Save the optimized solution γ and cost C_γ
17: **end for**
18: Find the minimum cost C_{γ^j} in $\{C_{\gamma^1}, ..., C_{\gamma^I}\}$
19: Output solution γ^j

Algorithm S4. Pseudocode of the solution initialization methods of the VLSNS solver.

The VLSNS solver provides two start solution initialization methods: the MSR method (Algorithm S4.1) and the MSG method (Algorithm S4.2). The MSR (multi-solution random) method generates multiple solutions at random. The MSG (multi-solution grid) method generates random solutions first and then equidistantly rolls the permutation order in each partite into multiple grid solutions for each random solution. n_k is the feature number of the k-th sample in a multipartite subgraph. I is the number of multi-start solutions.

Algorithm S4.1 Initialize random solutions for the VLSNS solver (MSR)

Input $\{n_1, \ldots, n_M\}$, I
Get max cardinality $p = max\{n_1, \ldots, n_M\} + 1$
Initialize solution set $\Gamma = \{\ \}$
for all $i \in \{1, \ldots, I\}$ **do**
    Initialize an ordered standard solution matrix γ with shape $p \times M$
    for all $m \in \{1, \ldots, M\}$ **do**
        Shuffle the permutation order of γ randomly in column m
    end for
    Add solution γ to Γ
end for
Output solution set Γ

Algorithm S4.2 Initialize grid solutions for the VLSNS solver (MSG)

Input $\{n_1, \ldots, n_M\}$, I
Get max cardinality $p = max\{n_1, \ldots, n_M\} + 1$
Initialize solution set $\Gamma = \{\ \}$
Initialize solution γ ←ordered standard solution with shape $p \times M$
for all $i \in \{1, \ldots, I\}$ **do**
    if i is a multiple of p **then**
        solution γ ←new random solution with shape $p \times M$
    end if
    for all $m \in \{1, \ldots, M\}$ **do**
        Roll the permutation order of γ in column m with shift $i \times m$
    end for
    Add solution γ to Γ
end for
Output solution set Γ

Figure S1. The RT drift distribution of library analytes on the TripleTOF 6600 dataset and the QE HF dataset.

Both the TripleTOF 6600 dataset and the QE HF dataset showed obvious drifts in the RT dimension, although they were acquired with different LC-MS platforms (the TripleTOF 6600 dataset was acquired by AB SCIEX TripleTOF 6600 interfaced with Shimazu L30A UPLC and the QE HF dataset was acquired by Thermo Q Exactive HF with Dionex UltiMate 3000 HPLC). We plotted the RT drift distributions of the library analytes across different samples below. The analytes can be divided into three groups according to the drift trends. One group had relatively stable retention time with sample acquisition, while the other two groups had opposite RT drift trends. The differences in RT drift trends proved that retention time alignment cannot be used alone for feature alignment, and proved the importance of feature matching. Also, the differences in RT drift trends made the TripleTOF 6600 dataset and the QE HF dataset more suitable for the evaluation of feature alignment methods.

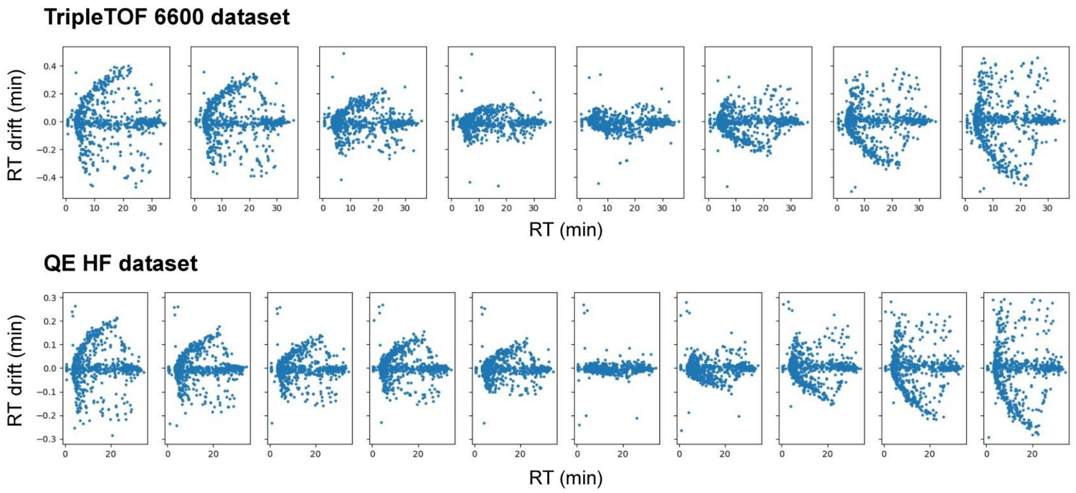

Table S1. Optimized parameters used in the TripleTOF 6600 dataset evaluation.

Methods	Step	Parameter	Value
MZmine 2 (version 2.53)	Raw data methods -> Feature detection -> Mass Detection	Mass detector	Wavelet transform
		Noise level	100
		Scale level	5
		Wavelet window size (%)	60
	Raw data methods -> Feature detection -> ADAP Chromatogram builder	Min group size in # of scans	5
		Group intensity threshold	200
		Min highest intensity	1000
		m/z tolerance	0.015 m/z or 0 ppm
	Feature list methods -> Feature detection -> Chromatogram deconvolution	Algorithm	Baseline cut-off
		m/z center calculation	MEDIAN
		Min peak height	1000
		Peak duration range (min)	0.08 - 10.00
		Baseline level	100
		Amplitude of noise	-
	Feature list methods -> Alignment -> RANSAC aligner	m/z tolerance	0.01 m/z or 0 ppm
		RT tolerance	0.5 min
		RT tolerance after correction	0.5 min
		RANSAC iterations	0 (auto)
		Minimum number of points	50 %
		Threshold value	0.5 min
		Linear model	not check
		Require same charge state	not check
XCMS (version 3.18.0)	Feature Detection	Method	centWave
		ppm	5
		minimum peak width	5
		maximum peak width	50
		mzdiff	0.015
		Signal/Noise threshold	5
		Integration method	1
		prefilter peaks	1
		prefilter intensity	200
		Noise filter	100
	Retention Time Correction	profStep	1
		missing	1
		extra	1
		smooth	loess
	Alignment	binSize	0.02
		bw	10
		minFraction	0.5
		minSamples	4
	Annotation	Search for	isotopes
		ppm	5
		m/z absolute error	0.015
OpenMS (version 2.7.0)	PeakPickerHiRes	signal_to_noise	0
		ms_levels	[ ]
		report_FWHM	false
		report_FWHM_unit	relative
		win_len	200
		bin_count	30
		min_required_elements	10
	FeatureFinderMetabo	noise_threshold_int	100
		chrom_peak_snr	3
		chrom_fwhm	10
		mass_error_ppm	10
		reestimate_mt_sd	true
		quant_method	area
		enabled	true
		width_filtering	fixed
		charge_lower_bound	1
		charge_upper_bound	3
		enable_RT_filtering	true
		isotope_filtering_model	none
		mz_scoring_13C	false
		report_convex_hulls	true
		remove_single_traces	false
		mz_scoring_by_elements	true
		elements	CHNOPSClFBr
	MapAlignerPoseClustering	ref_index	0
		max_num_peaks_considered	1000

		mz_pair_max_distance	0.01
		num_used_points	2000
		scaling_bucket_size	0.005
		shift_bucket_size	3
		second_nearest_gap	2
		use_identifications	false
		ignore_charges	true
		ignore_adduct	true
		rt_max_difference	30s
		mz_max_difference	0.01Da
	FeatureLinkerUnlabeledQT	use_identifications	false
		nr_partitions	100
		min_nr_diffs_per_bin	50
		min_IDscore_forTolCalc	1
		noID_penalty	0
		ignore_charge	true
		ignore_adduct	true
		rt_max_difference	30s
		mz_max_difference	0.01Da
**G-Aligner**	CoarseRegistration	solver	ransac
		centric_idx	0
		from_rt	0
		to_rt	inf
		mz_tolerance	0.01
		use_ppm	False
		rt_threshold	0.5
		rt_residual_threshold	0.05
		degree	1
	FineAssignment	rt_tolerance	0.3
		mz_tolerance	0.01
		use_ppm	False
		mz_factor	1
		rt_factor	1
		area_factor	1
		solver	gurobi / greedy / vlsns
		vlsns_solution_init_mode	msg / msr
		vlsns_solution_init_number	20
		vlsns_solution_update_mode	best

Table S2. Optimized parameters used in the QE HF dataset evaluation.

Methods	Step	Parameter	Value
**MZmine 2 (version 2.53)**	Raw data methods -> Feature detection -> Mass Detection	Mass detector	Wavelet transform
		Noise level	100
		Scale level	5
		Wavelet window size (%)	60
	Raw data methods -> Feature detection -> ADAP Chromatogram builder	Min group size in # of scans	5
		Group intensity threshold	1000
		Min highest intensity	100000
		m/z tolerance	0.005 m/z or 0 ppm
	Feature list methods -> Feature detection -> Chromatogram deconvolution	Algorithm	Noise amplitude
		m/z center calculation	MEDIAN
		Min peak height	100000
		Peak duration range (min)	0.08 - 10.00
		Baseline level	-
		Amplitude of noise	10000
	Feature list methods -> Alignment -> RANSAC aligner	m/z tolerance	0.005 m/z or 0 ppm
		RT tolerance	0.3 min
		RT tolerance after correction	0.3 min
		RANSAC iterations	0 (auto)
		Minimum number of points	50 %
		Threshold value	0.3 min
		Linear model	not check
		Require same charge state	not check
**XCMS (version 3.18.0)**	Feature Detection	Method	centWave
		ppm	5
		minimum peak width	5
		maximum peak width	50
		mzdiff	0.01
		Signal/Noise threshold	4
		Integration method	1
		prefilter peaks	1
		prefilter intensity	100000
		Noise filter	1000
	Retention Time Correction	profStep	1
		missing	1
		extra	1
		smooth	loess
	Grouping	binSize	0.01
		bw	6
		minFraction	0.5
		minSamples	5
**OpenMS (version 2.7.0)**	PeakPickerHiRes	signal_to_noise	0
		ms_levels	[ ]
		report_FWHM	false
		report_FWHM_unit	relative
		win_len	200
		bin_count	30
		min_required_elements	10
	FeatureFinderMetabo	noise_threshold_int	10000
		chrom_peak_snr	3
		chrom_fwhm	10
		mass_error_ppm	10
		reestimate_mt_sd	true
		quant_method	area
		enabled	true
		width_filtering	fixed
		charge_lower_bound	1
		charge_upper_bound	3
		enable_RT_filtering	true
		isotope_filtering_model	none
		mz_scoring_13C	false
		report_convex_hulls	true
		remove_single_traces	false

		mz_scoring_by_elements	true
		elements	CHNOPSClFBr
	MapAlignerPoseClustering	ref_index	0
		max_num_peaks_considered	1000
		mz_pair_max_distance	0.005
		num_used_points	2000
		scaling_bucket_size	0.005
		shift_bucket_size	3
		second_nearest_gap	2
		use_identifications	false
		ignore_charges	true
		ignore_adduct	true
		rt_max_difference	18s
		mz_max_difference	0.005Da
	FeatureLinkerUnlabeledQT	use_identifications	false
		nr_partitions	100
		min_nr_diffs_per_bin	50
		min_IDscore_forTolCalc	1
		noID_penalty	0
		ignore_charge	true
		ignore_adduct	true
		rt_max_difference	18s
		mz_max_difference	0.005Da
**G-Aligner**	CoarseRegistration	solver	ransac
		centric_idx	0
		from_rt	0
		to_rt	inf
		mz_tolerance	0.005
		use_ppm	False
		rt_threshold	0.3
		rt_residual_threshold	0.03
		degree	1
	FineAssignment	rt_tolerance	0.15
		mz_tolerance	0.003
		use_ppm	False
		mz_factor	1
		rt_factor	1
		area_factor	1
		solver	gurobi / greedy / vlsns
		vlsns_solution_init_mode	msg / msr
		vlsns_solution_init_number	20
		vlsns_solution_update_mode	best

Table S3. Optimized parameters used in the MTBLS562 dataset evaluation.

Methods	Step	Parameter	Value
MZmine 2 (version 2.53)	Raw data methods -> Feature detection -> Mass Detection	Mass detector<br>Noise level	Centroid<br>50
	Raw data methods -> Feature detection -> ADAP Chromatogram builder	Min group size in # of scans<br>Group intensity threshold<br>Min highest intensity<br>m/z tolerance	5<br>50<br>100<br>0.015 m/z or 0 ppm
	Feature list methods -> Feature detection -> Chromatogram deconvolution	Algorithm<br>m/z center calculation<br>Min peak height<br>Peak duration range (min)<br>Baseline level<br>Amplitude of noise	Baseline cut-off<br>MEDIAN<br>100<br>0.08 - 10.00<br>10<br>-
	Feature list methods -> Alignment -> RANSAC aligner	m/z tolerance<br>RT tolerance<br>RT tolerance after correction<br>RANSAC iterations<br>Minimum number of points<br>Threshold value<br>Linear model<br>Require same charge state	0.015 m/z or 0 ppm<br>0.3 min<br>0.3 min<br>0 (auto)<br>50 %<br>0.3 min<br>not check<br>not check
XCMS (version 3.18.0)	Feature Detection	Method<br>ppm<br>minimum peak width<br>maximum peak width<br>mzdiff<br>Signal/Noise threshold<br>Integration method<br>prefilter peaks<br>prefilter intensity<br>Noise filter	centWave<br>10<br>5<br>20<br>0.015<br>3<br>1<br>1<br>100<br>100
	Retention Time Correction	profStep<br>missing<br>extra<br>smooth	1<br>1<br>1<br>loess
	Alignment	binSize<br>bw<br>minFraction	0.02<br>10<br>0.5
OpenMS (version 2.7.0)	FeatureFinderMetabo	noise_threshold_int<br>chrom_peak_snr<br>chrom_fwhm<br>mass_error_ppm<br>reestimate_mt_sd<br>quant_method<br>enabled<br>width_filtering<br>charge_lower_bound<br>charge_upper_bound<br>enable_RT_filtering<br>isotope_filtering_model<br>mz_scoring_13C<br>report_convex_hulls<br>remove_single_traces<br>mz_scoring_by_elements<br>elements	100<br>1<br>6<br>10<br>true<br>area<br>true<br>fixed<br>1<br>2<br>true<br>none<br>false<br>false<br>false<br>false<br>CHNOPSClFBr
	MapAlignerPoseClustering	ref_index<br>max_num_peaks_considered<br>mz_pair_max_distance<br>num_used_points<br>scaling_bucket_size<br>shift_bucket_size<br>second_nearest_gap<br>use_identifications<br>ignore_charges<br>ignore_adduct<br>rt_max_difference<br>mz_max_difference	0<br>1000<br>0.015<br>2000<br>0.005<br>3<br>2<br>false<br>true<br>true<br>30s<br>0.015Da
	FeatureLinkerUnlabeledQT	use_identifications<br>nr_partitions	false<br>100

		min_nr_diffs_per_bin	50
		min_IDscore_forTolCalc	1
		noID_penalty	0
		ignore_charge	true
		ignore_adduct	true
		rt_max_difference	30s
		mz_max_difference	0.015Da
**G-Aligner**	CoarseRegistration	solver	ransac
		centric_idx	0
		from_rt	0
		to_rt	inf
		mz_tolerance	0.01
		use_ppm	False
		rt_threshold	0.3
		rt_residual_threshold	0.05
		degree	1
	FineAssignment	rt_tolerance	0.1
		mz_tolerance	0.01
		use_ppm	False
		mz_factor	1
		rt_factor	1
		area_factor	1
		solver	gurobi / greedy / vlsns
		vlsns_solution_init_mode	msg / msr
		vlsns_solution_init_number	20
		vlsns_solution_update_mode	best

Table S4. Time cost on manually annotated feature sets of the TripleTOF 6600 dataset, the QE HF dataset and the MTBLS562 dataset.

The acceleration ratio is the computation speed improvement ratio of the VLSNS solver compared with the Gurobi solver.

Dataset	Method	Time cost (s)	Acceleration ratio
TripleTOF 6600 dataset (8 files, 12075 features)	MZmine2 RANSAC	13	-
	OpenMS QT	0.1	-
	XCMS Group	4.3	-
	XCMS OBI-Warp	131	-
	Local bipartite	16	-
	G-Aligner Greedy	137	-
	G-Aligner Gurobi	153	-
	G-Aligner VLSNS_MSR	74	2.1
	G-Aligner VLSNS_MSG	73	2.1
QE HF dataset (10 files, 24541 features)	MZmine2 RANSAC	35	-
	OpenMS QT	0.2	-
	XCMS Group	6	-
	XCMS OBI-Warp	312	-
	Local bipartite	39	-
	G-Aligner Greedy	369	-
	G-Aligner Gurobi	413	-
	G-Aligner VLSNS_MSR	162	2.5
	G-Aligner VLSNS_MSG	161	2.6
MTBLS562 dataset (40 files, 12912 features)	MZmine2 RANSAC	26	-
	OpenMS QT	0.1	-
	XCMS Group	3	-
	XCMS OBI-Warp	2060	-
	Local bipartite	13	-
	G-Aligner Greedy	623	-
	G-Aligner Gurobi	685	-
	G-Aligner VLSNS_MSR	46	13.5
	G-Aligner VLSNS_MSG	45	13.8

Table S5. Time cost on software self-extracted feature sets of the TripleTOF 6600 dataset, the QE HF dataset and the MTBLS562 dataset.

The acceleration ratio is the computation speed improvement ratio of the VLSNS solver compared with the Gurobi solver.

Dataset	Feature set	Method	Time cost (s)	Acceleration ratio
TripleTOF 6600 dataset (8 files)	MZmine 2 (170788 features)	MZmine2 RANSAC	151	-
		Local bipartite	58	-
		G-Aligner Greedy	654	-
		G-Aligner Gurobi	755	-
		G-Aligner VLSNS_MSR	312	2.4
		G-Aligner VLSNS_MSG	273	2.8
	OpenMS (420566 features)	OpenMS QT	53	-
		Local bipartite	103	-
		G-Aligner Greedy	3254	-
		G-Aligner Gurobi	3824	-
		G-Aligner VLSNS_MSR	1379	2.4
		G-Aligner VLSNS_MSG	1364	2.4
	XCMS (463629 features)	XCMS Group	504	-
		XCMS OBI-Warp	152	-
		Local bipartite	100	-
		G-Aligner Greedy	2474	-
		G-Aligner Gurobi	2905	-
		G-Aligner VLSNS_MSR	952	3.1
		G-Aligner VLSNS_MSG	937	3.1
QE HF dataset (10 files)	MZmine 2 (160843 features)	MZmine2 RANSAC	111	-
		Local bipartite	54	-
		G-Aligner Greedy	1002	-
		G-Aligner Gurobi	1139	-
		G-Aligner VLSNS_MSR	234	4.9
		G-Aligner VLSNS_MSG	224	5.1
	OpenMS (302100 features)	OpenMS QT	150	-
		Local bipartite	75	-
		G-Aligner Greedy	3675	-
		G-Aligner Gurobi	4133	-
		G-Aligner VLSNS_MSR	448	9.2
		G-Aligner VLSNS_MSG	431	9.6
	XCMS (384020 features)	XCMS Group	156	-
		XCMS OBI-Warp	438	-
		Local bipartite	142	-
		G-Aligner Greedy	2124	-
		G-Aligner Gurobi	2334	-
		G-Aligner VLSNS_MSR	577	4.0
		G-Aligner VLSNS_MSG	562	4.2
MTBLS562 dataset (40 files)	MZmine 2 (2407090 features)	MZmine2 RANSAC	25101	-
		Local bipartite	1680	-
		G-Aligner VLSNS_MSR	19081	-
		G-Aligner VLSNS_MSG	19503	-
	OpenMS	OpenMS QT	88	-

(1416524 features)	Local bipartite	630	-
	G-Aligner VLSNS_MSR	10984	-
	G-Aligner VLSNS_MSG	10865	-
XCMS	XCMS Group	1399	-
(868052 features)	XCMS OBI-Warp	467	-
	Local bipartite	481	-
	G-Aligner VLSNS_MSR	3557	-
	G-Aligner VLSNS_MSG	2825	-

Appendix S1. Data annotation procedure in MetaPro.

1. Prepare dataset in vendor formats.
2. Convert vendor format to Aird format with AirdPro (https://github.com/CSi-Studio/AirdPro).
3. Place the converted file in the data repository of MetaPro, such as /data/projectName/batchName/files.aird
4. Create a project in MetaPro with the same projectName in data repository.
5. Scan files to the project
6. Upload internal standard library and analyte library. The analyte library should contain the compound name, m/z value and RT value of the analytes provided along with the dataset. Internal standards can be chosen from the analytes, which should have high intensity and no noise peak in extracted ion chromatogram. Internal standard library is used for retention time alignment.
7. Set analysis parameters.
8. Perform targeted extraction on the internal standard library and analyte library.
9. Perform manual batch inspection on the targeted analyzed data of the analyte library to make sure the right peak is accurately integrated and identified. When an analyte has no peaks or noisy peaks in most of the runs, set the status of the analyte to failed. Otherwise, set the analyte status to succeed.
10. Export the annotated identified features of succeed analytes to a result table in xlsx format.
